# A multiscale approach for the reconstruction of the fiber architecture of the human brain based on 3D-PLI

**DOI:** 10.3389/fnana.2015.00118

**Published:** 2015-09-03

**Authors:** Julia Reckfort, Hendrik Wiese, Uwe Pietrzyk, Karl Zilles, Katrin Amunts, Markus Axer

**Affiliations:** ^1^Institute of Neuroscience and Medicine (INM-1), Structural and Functional Organization of the Human Brain, Research Centre JülichJülich, Germany; ^2^Institute of Neuroscience and Medicine (INM-4), Medical Imaging Physics, Research Centre JülichJülich, Germany; ^3^Department of Mathematics and Natural Sciences, University of WuppertalWuppertal, Germany; ^4^Department of Psychiatry, Psychotherapy and Psychosomatics, University Hospital, RWTH Aachen University and JARA Translational Brain MedicineAachen, Germany; ^5^Cécile and Oskar Vogt Institute for Brain Research, Heinrich-Heine University DüsseldorfDüsseldorf, Germany

**Keywords:** polarized light imaging, brain, fiber orientation, multiscale approach, connectome

## Abstract

Structural connectivity of the brain can be conceptionalized as a multiscale organization. The present study is built on 3D-Polarized Light Imaging (3D-PLI), a neuroimaging technique targeting the reconstruction of nerve fiber orientations and therefore contributing to the analysis of brain connectivity. Spatial orientations of the fibers are derived from birefringence measurements of unstained histological sections that are interpreted by means of a voxel-based analysis. This implies that a single fiber orientation vector is obtained for each voxel, which reflects the net effect of all comprised fibers. We have utilized two polarimetric setups providing an object space resolution of 1.3 μm/px (microscopic setup) and 64 μm/px (macroscopic setup) to carry out 3D-PLI and retrieve fiber orientations of the same tissue samples, but at complementary voxel sizes (i.e., scales). The present study identifies the main sources which cause a discrepancy of the measured fiber orientations observed when measuring the same sample with the two polarimetric systems. As such sources the differing optical resolutions and diverging retardances of the implemented waveplates were identified. A methodology was implemented that enables the compensation of measured different systems' responses to the same birefringent sample. This opens up new ways to conduct multiscale analysis in brains by means of 3D-PLI and to provide a reliable basis for the transition between different scales of the nerve fiber architecture.

## 1. Introduction

Decoding the human brain is one of the major targets for neuroscientists and researchers around the world. To understand the function of the brain and clinical symptoms in patients with neuropsychiatric disorders, it is indispensable to study its underlying structure, i.e., the organization of neurons and their intricate connections. The mapping of the nerve fiber architecture of the brain is a multiscale challenge as the size of the structures range from a few nanometers for the diameter of microfilaments to several centimeters for long distance inter-hemispheric fibers.

Over the past century several imaging techniques were developed to address the brain's fiber architecture, ranging from macroscopic techniques (e.g., dissection, Klingler, 1935; Tüere et al., 2000; diffusion tensor imaging, Basser et al., 1994; Pierpaoli and Basser, 1996; Conturo et al., 1999; Beaulieu, 2002; Mori and Zhang, 2006; Schmahmann et al., 2007; Johansen-Berg and Rushworth, 2009; high angular resolution imaging methods, Tuch et al., 2002; Jansons and Alexander, 2003; Tournier et al., 2004, 2007; Behrens et al., 2007; Dell'Acqua et al., 2007; Descoteaux et al., 2007) to microscopic techniques (classical myelin staining, Bürgel et al., 1997, 2006; polarization-sensitive optical coherence tomography, de Boer et al., 1997; Wang et al., 2011; knife-edge scanning microscopy, Li et al., 2010; or light-sheet fluorescence microscopy, Silvestri et al., 2012).

So far, it is difficult or even impossible to integrate most of the multimodal imaging data sets. This is caused by the lack of tools to bridge different scales and types of structural descriptions, thus, preventing the investigation of the organization of the brain at different levels of detail. A solution of such a problem is to realize a “multiscale approach” with imaging setups that provide complementary resolutions, and rely on the same imaging technique.

3D-Polarized Light Imaging (3D-PLI, Axer et al., 2011a) allows to investigate fiber tracts micro- and macroscopically, depending on the optical setup. Using 3D-PLI, it is possible to derive unit vectors that describe the orientation of fiber tracts and single nerve fibers in histological sections of postmortem brains. The potential to investigate nerve fibers by means of polarimetry is known for more than a century (Brodmann, 1903; Goethlin, 1913; Schmidt, 1923; Schmitt and Bear, 1937; Wolman, 1975), but only the recent advances in digital image acquisition, 3D reconstruction, and big data processing has enabled the systematic investigation of the 3D orientation of nerve fibers (Axer et al., 2011b; Amunts et al., 2013b). Utilization of high performance computing tools and techniques paved the way toward whole brain analyses (Amunts et al., 2013a). As a result, 3D-PLI enables unique whole brain studies in rodents, monkeys, and even humans with reasonable efforts in laboratory work and infrastructure within affordable time frames. Nonetheless, the complexity of the entire human brain is still a challenge and mapping its fiber architecture will benefit significantly from the multiscale approach presented here as it provides a rational and accessible merger of context and detail.

In the present study, two custom-made polarimeters with different optical resolutions and sensitivities optimized for 3D-PLI measurements were employed to establish a multiscale approach, the polarizing microscope (PM) and the large-area polarimeter (LAP). Each system offers unique features (Axer et al., 2011b): The PM enables mosaic-like scanning of sections at microscopical resolution, thus, even enabling the detection of single fibers within the different layers of the cerebral cortex. A large single human brain section which is scanned in up to 4880 tiles requires about 21 h scanning time, resulting in a data volume of 703 GB. The LAP allows to image a whole human brain section within a single shot. The imaging procedure consists of the imaging of the section from five different view angles which takes about 15 min of scanning time and generates a data amount of 3 GB per section. The fast acquisition time is a prerequisite to map long-distance white matter tracts of complete human brains. A strategy to circumvent parts of the methodical challenges of the PM is to combine the information gained with both systems. Moreover, such combination of the two systems with different optical resolutions closes the gap between different spatial scales. This requires that the fiber orientations measured with both systems are comparable. Previous studies revealed that the systems differ in their properties resulting in different system responses to the same sample (Reckfort et al., 2013). Therefore, the purpose of this study was to develop a methodology to compensate these differences by means of corrections applicable to image analysis and signal interpretation for PM and LAP.

## 2. Materials and methods

### 2.1. Principles of 3D-polarized light imaging

3D-Polarized Light Imaging (Axer et al., 2011a,b) utilizes the optical birefringence of nerve fibers that is attributed to the highly ordered molecular organization of the lipids and proteins building the myelin sheaths surrounding axons (Martenson, 1992). In 3D-PLI, a polarimetric setup enables the detection of birefringent structures and the determination of their spatial orientation within a given sample. For this purpose, the brain tissue has to be cut into 70 μm thin unstained histological sections (cf. Section 2.4) to achieve transmissive imaging in the visible spectrum.

**Figure 1:**
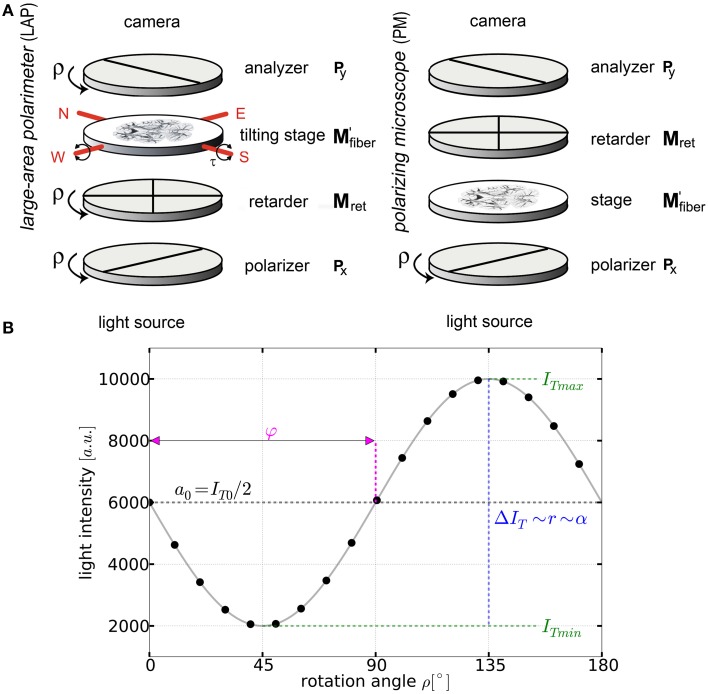
**Basic principles of 3D-PLI**. **(A)** Schemes of the large-area rotating polarimeter with a tilting stage and of the polarizing microscope (PM) from Taorad GmbH (Germany). **(B)** The signal intensity profile depending on the rotation angle ρ of the filters for a single pixel measured with a polarimeter. To obtain the fiber orientation, the measured light intensities are studied pixel-wise as a function of discrete rotation angles. The derived physical model provides (continuous black line) and relates the sine phase to the direction φ and the amplitude to the inclination angle α.

Both polarimetric setups employed in this study are composed of three optical filters (two crossed linear polarizers and one quarter waveplate with its fast axis adjusted at an angle of 45° with respect to the transmission axes of the first linear polarizer) to control the polarization state of the incident light and to analyze its changes after interacting with the nerve fibers of the tissue section (Figure 1A).

By rotating at least one of the linear polarizers in equidistant angles (i.e., Δρ = 10°) within the range of ρ = 0° and 170° a stack of 18 images is acquired. The measured intensity profile for an individual pixel across the stack of images describes a sinusoidal curve that depends on the orientation of fibers within this pixel (Figure 1B). The physical description of the light intensity profile is derived by the Jones matrix calculus (Larsen et al., 2007), as the system is composed of linear optical elements and the light is considered fully polarized:

(1) $$I_{T}(\rho )=\frac{I_{T0}}{2}\left[1+\sin(2\rho -2\varphi )r\right]$$

with

(2) $$r=\left|\sin\left(2\pi \frac{t\Delta n}{\lambda }\cos^{2}\alpha \right)\right|,$$

where *I*_*T*_(ρ) describes the measured light intensity for the filters rotated by an angle of ρ and *I*_*T*0_ reflects the attenuated light intensity. The retardation *r* indicates the strength of birefringence and is quantified by the amplitude of the measured signal normalized by *I*_*T*0_. The retardation *r* depends on the wavelength λ of the light source, the birefringence of the tissue Δ*n*, the section thickness *t*, and the inclination angle α describing the fiber elevation with respect to the sectioning plane.

Equation (1) can be parameterized by means of discrete harmonic Fourier analysis (Glazer et al., 1996; Axer et al., 2011b) as follows:

(3) $$I_{T}(\rho )=a_{0}+a_{1}\sin(2\rho )+b_{1}\cos(2\rho ),$$

with

(4) $$a_{0}=\frac{I_{T0}}{2},\;a_{1}=\frac{I_{T0}}{2}r\cos(2\varphi ),\;b_{1}=-\frac{I_{T0}}{2}r\sin(2\varphi ).$$

These coefficients are computed from the measured intensity profile *I*_*T*_(ρ_*i*_) in each individual pixel by

(5) $$\begin{array}{l} a_{0}=\frac{1}{N}\sum_{i=1}^{N}I_{T}(\rho_{i}),\;a_{1}=\frac{2}{N}\sum_{i=1}^{N}I_{T}(\rho_{i})\sin(2\rho_{i}),\\ \;b_{1}=\frac{2}{N}\sum_{i=1}^{N}I_{T}(\rho_{i})\cos(2\rho_{i}), \end{array}$$

where ρ_*i*_ denotes the filter rotation angle and *N*(= 18) the total number of sampled data points.

Combining the Fourier coefficients, it is possible to retrieve light retardation, light transmittance and to quantify the fiber orientation (α,φ) for each image pixel:

(6) $$\mathrm{Transmittance}:\;I_{T0}=2a_{0},$$

(7) $$\mathrm{Retardation}:\;\;r={\left(a_{1}^{2}+b_{1}^{2}\right)}^{\frac{1}{2}}/a_{0},$$

(8) $$\mathrm{Direction}:\;\;\;\;\;\varphi =\frac{1}{2}\text{arctan}2(b_{1},-a_{\text{1}})+\frac{\pi }{\text{2}}=\frac{1}{2}\arg\;(a_{1}+ib_{1})$$

The fiber inclination angle α can be extracted from Equation (2), though it has to be noted that it is not possible to determine the sign of the inclination angle with a single planar measurement. To resolve the ambiguity of the sign of the inclination angle the specimen stage has to be tilted. For more detail please refer to (Kleiner et al., 2012; Wiese et al., 2014). Furthermore, to determine the fiber inclination angle the wavelength, the tissue birefringence and the section thickness have to be known precisely. As it is difficult to determine these parameters individually, they have been merged to a single parameter referred to as relative thickness *t*_rel_ as introduced by Axer et al. (2011a). The relative thickness is defined as the ratio of the actual section thickness *t* and the section thickness at which a fiber that runs parallel to the sectioning plane (i.e., α ≈ 0°) acts as an ideal quarter waveplate. This approach leads to

(9) $$r=\left|\sin\left(\frac{\pi }{2}t_{\text{rel}}\cos^{2}\alpha \right)\right|.$$

Equation (9) enables the determination of *t*_rel_ by means of reference measurements in brain sections with fiber bundles that have been cut along their principal paths (i.e., α≈0°). Both direction angle φ and inclination angle α constitute a unit vector that describes the local spatial fiber orientation. The image of all derived fiber orientations covering an entire brain section represents the fiber orientation map (FOM, Axer et al., 2011a).

### 2.2. Polarimetric equipment

In order to realize the multiscale approach, two polarimeters (cf. Figure 1A) were used in the present study: the polarization microscope (PM) and the large-area polarimeter (LAP, Axer et al., 2011b). In the LAP, the brain section is illuminated with circularly polarized light. In this setup, all optical filters are rotated simultaneously during the measurement. In contrast, the light path through the filters is reversed in the PM and only the polarizer is rotated. The light intensity profiles obtained with both setups, however, can be described by the same equation (Equation 1) which enables the employment of the same strategy for data analysis.

#### 2.2.1. LAP

The LAP is equipped with a quarter waveplate optimized for λ_ret_ = 560nm and an LED light source emitting at λ_ill_ = (525±25)nm. It has specifically been designed to digitize large histological sections (~200cm^2^, i.e., whole human brain sections) in single shot acquisitions. Moreover, the LAP has a tilting specimen stage providing tilt angles of |τ|≤8°. This feature has been introduced to compensate for intrinsic limitations of planar projection imaging technologies, such as the inclination sign ambiguity (Kleiner et al., 2012; Wiese et al., 2014). By assigning a definite preference to the inclination angle, the tracking of fiber bundles across neighboring brain sections becomes feasible. The optical resolution limit according to the Rayleigh criterion was determined to be 159 μm and the object space resolution to be 64 μm/px (Reckfort et al., 2013). Based on repeated measurements of the same tissue sample, the variances of the measured retardation and direction values were extracted to be σ(*r*_LAP_) = 0.008 and σ(φ_LAP_) = 0.36°, respectively.

#### 2.2.2. PM

In the PM, a quarter waveplate with an optimal working wavelength of λ_ret_ = 545nm and an LED light source with λ_ill_ = (543.5±10)nm as central peak wavelength, are implemented. The determined optical resolution limit is 3.9 μm and the object space resolution is 1.33 μm/px (Reckfort et al., 2013). The PM has a limited field of view of (2.7 × 2.7)mm^2^ and the brain sections are scanned stepwise with an overlapping field of view of 30% to enable robust stitching of the neighboring images to create a consistent image of the whole section. In total, a whole brain section is build of up to 4880 single tiles and takes about 21 h scanning time. The PM's imaging procedure is limited to planar measurements, i.e., no tilting of the specimen stage is possible. Hence, the current PM setup does not provide an unambiguous sign for the fiber inclination angle. The variances of the measured retardation and direction values were extracted from repeated measurements of the same object to be σ(*r*_PM_) = 0.007 and σ(φ_PM_) = 0.29°, respectively.

### 2.3. Influence of the wavelength discrepancy

A previous study by Reckfort et al. (2013) indicated that the discrepancy of the illumination peak wavelength and the design wavelength of the quarter-waveplate used in the LAP influences the response of the system to birefringent samples. The current analysis (cf. Equations 1–8) assumes that the light is retarded by a quarter-wavelength by the used waveplate. As for the LAP setup the retardance (i.e., phase retardation) induced by the waveplate for the wavelength of the employed illumination source has not been specified by the manufacturer, the data analysis described in Section 2.1 had to be adapted. For this purpose, the derivation of the transmitted light intensity by means of the Jones calculus was generalized in such a way that the retardance γ induced by the waveplate is arbitrary.

To describe the optical setup the Jones matrices *P*_*x*_ and *P*_*y*_ denote the implemented linear polarizers and the matrix *M*_ret_(γ) the waveplate inducing an arbitrary retardance. Further the brain tissue is represented by a Jones matrix for a quarter-wave retarder with an arbitrary rotation angle and retardance.

(10) $${\vec{E}}_{T}{}^{\prime }(\delta ,\beta ,\gamma )=P_{y}\cdot {M^{\prime }}_{\text{fiber}}(\delta ,\beta )\cdot M_{\text{ret}}(\gamma )\cdot P_{x}\cdot \vec{E}.$$

When an arbitrary retardance induced by the waveplate is assumed, the intensity profile of the current setup is described, similarly to Equation (3), by a Fourier series with the coefficients *a*_0_, *a*_1_, *b*_1_, *a*_2_, and *b*_2_^1^. This enables a precise description of the measured intensity profile.

(11) $$I_{T}^{\prime }(\rho )=a_{0}^{\prime }+a_{1}^{\prime }\sin(2\rho )+b_{1}^{\prime }\cos(2\rho )+a_{2}^{\prime }\sin(4\rho )+b_{2}^{\prime }\cos(4\rho ),$$

with the Fourier coefficients:

$$\begin{array}{l} a_{0}^{\prime }=\frac{I_{T0}^{\prime }}{2}\sin^{2}\left(\delta /2\right)+I_{T0}^{\prime }\sin^{2}(\gamma /2)\cos^{2}\left(\delta /2\right),\\ a_{1}^{\prime }=\frac{I_{T0}^{\prime }}{2}\sin(\gamma )\sin(\delta )\cos(2\varphi ),\\ b_{1}^{\prime }=-\frac{I_{T0}^{\prime }}{2}\sin(\gamma )\sin(\delta )\sin(2\varphi ),\\ a_{2}^{\prime }=-\frac{I_{T0}^{\prime }}{2}\cos(\gamma )\sin^{2}\left(\delta /2\right)\cos(4\varphi ),\\ b_{2}^{\prime }=-\frac{I_{T0}^{\prime }}{2}\cos(\gamma )\sin^{2}\left(\delta /2\right)\sin(4\varphi ). \end{array}$$

The new determined Fourier coefficients differ from the ones determined for the ideal case of a phase retardance of γ = π/2 (cf. Equation 4). If the light retardance differs from the ideal case, additional coefficients *a*′_2_ and *b*′_2_ have to be considered. The fiber direction angle (phase of sinus) can be calculated as before by (cf. Equation 4):

(12) $$\varphi^{\prime }=\frac{1}{2}\arctan2(b_{1}^{\prime },-a_{1}^{\prime })+\frac{\pi }{2}.$$

In contrast, the retardation and transmittance differ as compared to the original description which considers a retardance induced by a quarter waveplate. It is possible to derive the retardation *r*′ and transmittance *I*′_*T*0_ by employing all five of the new Fourier coefficients:

(13) $$\begin{array}{l} r^{\prime }=\sqrt{a\prime_{1}^{2}+b\prime_{1}^{2}}\cdot \;\frac{2\sin^{2}(\gamma /2)}{\left|\sin(\gamma )\right|}(a_{0}^{\prime }-\frac{\sqrt{a\prime_{2}^{2}+b\prime_{2}^{2}}}{\left|\cos(\gamma )\right|}\\ \;{\left(1-2\sin^{2}(\gamma /2)\right)))}^{-1}, \end{array}$$

(14) $$I\prime_{T0}=\frac{1}{\sin^{2}(\gamma /2)}\left(a_{0}^{\prime }-\frac{\sqrt{a\prime_{2}^{2}+b\prime_{2}^{2}}}{\left|\cos(\gamma )\right|}(1-2\sin^{2}(\gamma /2))\right)$$

The dependency of *r*′ and *I*′_*T*0_ on the Fourier components are more complex as compared to the ideal quarter-wave retardance. Therefore, it is no longer possible to assign the measured relative amplitude to the retardation and the average intensity across all 18 measurements to the transmittance.

### 2.4. Tissue preparation

**Figure 2:**
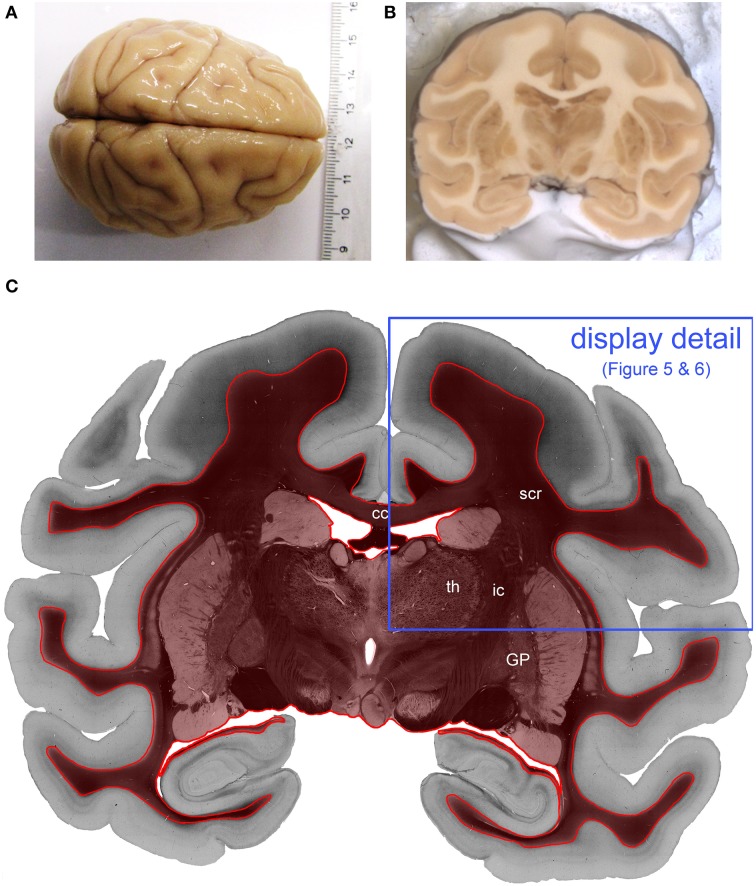
**Processed vervet monkey brain**. **(A)** Shows the whole vervet monkey brain before sectioning and **(B)** shows the a blockface image which is captured during the cutting procedure and **(C)** is the transmittance image of the evaluated coronal, vervet monkey brain section. The red label indicates the evaluated region of interest. The blue frame indicates the detail of the section which is displayed in **Figures 4**, **5**. (cc, corpus callosum; GP, globus pallidus; ic, internal capsule; scr, superior corona radiata; th, thalamus.)

The influence of the different system properties on tissue measurements was investigated in coronal sections of a postmortem vervet/African green monkey brain (*Chlorocebus aethiops sabaeus)* (Figures 2A,B). The brain was retrieved through the NIH program *1R01MH092311-01A1* in accordance with legal and ethical requirements. The vervet monkey was a member of the Vervet Research Colony (VRC) at Wake Forest University. The Wake Forest University complies with the Principles for Use of Animals, the Guide for the Care and Use of Laboratory Animals, and all provisions of the Animal Welfare Act. The project was approved by the UCLA Chancellor's Animal Research Committee (ARC) ARC #2011-135 and by the Wake Forest Institutional Animal Care and Use Committee IACUC #A11-219.

The vervet monkey brain was perfusion fixed in 4% buffered paraformaldehyde, cryo-protected with a 20% glycerin solution and deep frozen at −70°C. The brain was serially sectioned in the coronal plane (section thickness: 70 μm) employing a cryostat microtome (Polycut CM 3500, Leica, Germany), embedded with a glycerin solution and coverslipped. Afterwards, the sections were immediately measured with the LAP and the PM.

### 2.5. Correlation between LAP and PM measurements

To evaluate the consistency of the fiber orientations obtained with the PM and the LAP, one exemplary coronal section of the vervet monkey brain has been selected and processed here.

For a pixel-wise comparison, the stitched PM image (44, 517 × 34, 024 px) was downsampled to match the image resolution and dimension of the LAP data set (937 × 716 px). This was done by means of a circular Gaussian filter. The standard deviation σ = 20px ~ 159/3.9 μm was chosen to replicate the lower optical resolution of the LAP. Furthermore, the filtered images were scaled by a factor of 0.0208 = 1.33/64, which corresponds to the ratio of the object space resolutions of both systems and reproduces the spatial sampling of the CCD camera implemented in the LAP. As a result, one downsampled pixel value corresponds to the average value of approximately 48 × 48 px of the Gaussian filtered PM image. These two steps of downsampling were performed with the open source image processing software *Fiji* (Schindelin et al., 2012).

The downsampling was applied directly to the maps of the Fourier coefficients [*a*_0_, *a*_1_, and *b*_1_ (cf. Equation 3)]. This approach reproduces the averaging process of the sinusoidal signals during the imaging process. Based on the downsampled Fourier coefficient maps, the downsampled direction and retardation maps were calculated according to Equation (4). The direction values determined with the PM were corrected by adding a value of 20.63° as the x-axis of the first linear polarizer is rotated by this value.

The LAP image was non-linearly registered to the PM image to ensure pixel-precise comparison. This is necessary as a cryo-section embedded in glycerin and coverslipped can still undergo slight deformations or movements. However, the changes are very slow compared to the duration of a single measurement. Still, to ensure the best possible comparison a non-linear registration was performed to align both images. The images were subdivided into four regions of interest (ROI) to minimize the influence of local rectifications. The images were pre-registered using a rigid and affine transformation. The registration was further improved by applying a b-spline transformation (Yoo et al., 2002). All registration steps were based on the toolbox elastix (Klein et al., 2010).

The retrieved fiber direction angle and retardation maps measured with the LAP and the downsampled PM were evaluated pixel-wise by means of difference images and scatter plots. For a correct interpretation of the difference map based on the measured direction, the represented data needs to be filtered to account for the periodicity of the parameter space of the direction angle. When analyzing the discrepancy between the direction of two fiber orientations, it has to be ensured that always the angle smaller than 90° is retrieved. This is realized by subtracting 180° from all values larger than Δφ = 90° and to add 180° to all values lower than Δφ = 90° in the differential map. The analysis was based on the evaluation of the white matter and basal ganglia (see red rimmed area in Figure 2). In total 206,788 px single pixels in the white matter were evaluated. The scatterplots were fitted based on a simple linear regression.

To investigate the consequences of the newly proposed evaluation analysis of the measured signals, the data obtained with the LAP was evaluated employing a retardance of γ_1_ = 0.25π and γ_2_ = 0.259π. The determined retardation values were further compared to measurements of the PM.

### 2.6. Influence of the lateral partial volume effect

In order to realize a multiscale approach for connectivity studies the influence of the different optical resolutions of the employed polarimetric systems is considered. These different resolutions affect particularly measurements of heterogeneous tissue (e.g., regions which contain crossing fibers).

If a single volume element (voxel) contains only a single fiber, the measured intensity profile within the corresponding pixel will encode the spatial fiber orientation. For a coarser optical resolution the signals of multiple pixels (in the image-plane) are averaged and cause the “lateral partial volume effect.” Depending on the arrangement of fibers, which are measured in the same pixel, it is difficult to decode the information about multiple fiber orientations from a single sinusoidal signal. The more dispersed the fiber orientations are, the more pronounced is the partial volume effect. The lateral partial volume effect in 3D-PLI depends on the inhomogeneity of biological tissue and the employed spatial resolution with which these structures are imaged. Knowing the magnitude of the local lateral partial volume effect improves the interpretation of 3D-PLI measurements. A suitable measure for this magnitude can be derived by analyzing the averaging of the signals in the downsampling procedure. To understand this process, the averaging of the sinusoidal signals of two individual pixels (cf. Equation 1)

$$\begin{array}{l} I_{T1}(\rho )=\frac{I_{T0}}{2}(1+r_{1}\sin(2\rho -2\varphi_{1})),\\ I_{T2}(\rho )=\frac{I_{T0}}{2}(1+r_{2}\sin(2\rho -2\varphi_{2})), \end{array}$$

is examined. The arithmetic mean of two sinusoidal curves results in a new sinusoidal curve with an amplitude $\tilde{r}$ and a phase $\tilde{\varphi }$:

(15) $$\frac{I_{T1}(\rho )+I_{T2}(\rho )}{2}=\frac{I_{T0}}{2}(1+\tilde{r}\sin(2\rho -2\tilde{\varphi })),$$

with

(16) $$\tilde{r}=\frac{I_{T0}}{2}\sqrt{r_{1}^{2}+r_{2}^{2}+2r_{1}r_{2}\cos(2\varphi_{1}-2\varphi_{2})}\leq \frac{r_{1}+r_{2}}{2}$$

and

(17) $$\tilde{\varphi }=\frac{1}{2}\text{arctan }\left(\frac{r_{1}\sin(2\varphi_{1})-r_{2}\sin(\varphi_{2})}{r_{1}\cos(2\varphi_{1})+r_{2}\cos(\varphi_{2})}\right).$$

The difference between the averaged amplitude ($\bar{r}=\left(r_{1}+r_{2}\right)∕2$) and the amplitude of the averaged sinusoidal curves ($\tilde{r}$) increases when the difference of the in-plane direction (correlated to the phase difference) of the two fibers increases. Hence, $\bar{r}-\tilde{r}$ can be used to quantify the heterogeneity of the tissue. For example, for parallel fibers, the averaging of the amplitudes of each pixel gives the same results as when the sinusoidal curves are averaged. As soon as the alignment of the fibers is heterogeneous, the averaged amplitude value is higher than the amplitude value of the averaged sinusoidal signals.

In order to test the hypothesis that the difference between the averaged amplitude value and the amplitude of the averaged sinus curves is a suited measure for the quantification of the lateral partial volume effect, the downsampled PM data was compared to LAP data. For this purpose, the PM data was downsampled by applying a Gaussian filter and scaling factor (σ_gauss_ = 20, *f*_sc_ = 1/48) to the retardation map based on the original PM measurements. This represents an averaging of the amplitudes, while the LAP measurement represents the averaging of the sinusoidal signals. Then the two retardation maps were subtracted from each other.

## 3. Results

### 3.1. Correlation between LAP and PM measurements

**Figure 3:**
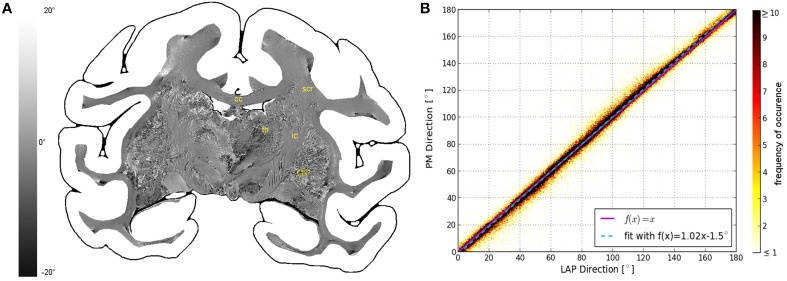
**Consistency of the measured fiber direction angle**. **(A)** Displays a differential map of the fiber direction maps obtained with the PM and LAP. It highlights in which areas the largest differences of the measured fiber directions occur. **(B)** Shows a scatterplot displaying a direct comparison of the measured fiber direction angles. The color code indicates the number of occurrences.

**Figure 4:**
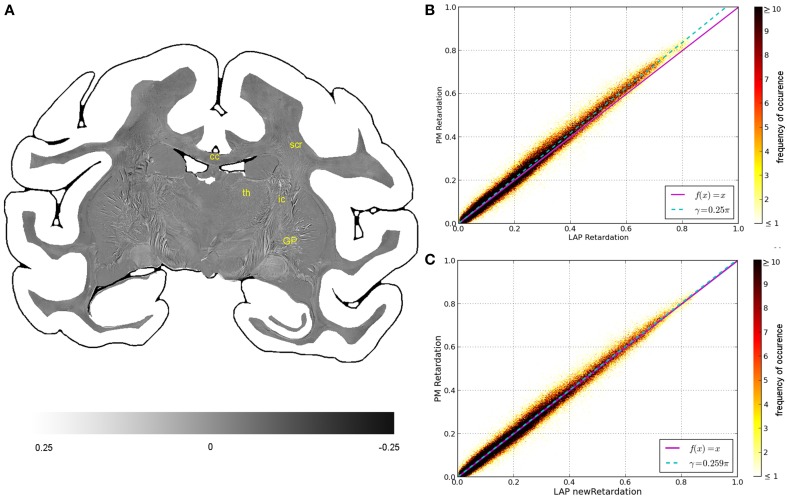
**Consistency of the measured retardation value**. **(A)** Shows a differential map obtained from subtracting the retardation map measured with the LAP from the downsampled PM retardation map. Bright pixels highlight in which areas the largest differences between the two maps occur. **(B,C)** Are scatterplots which show a direct comparison of the measured retardation values. For both scatterplots the same PM data were used while the analysis of the LAP measurements differ. For **(B)** the LAP retardation values are derived employing a retardance of γ = 0.25π and for **(C)** a retardance of γ = 0.259π was used. The color code indicates the number of occurrences.

The fiber direction angles and retardation values measured with the PM and the LAP were compared in scatterplots (cf. Figures 3B, 4B,C). The scatterplot in Figure 3B shows the correlation between the value of the in-plane fiber direction measured with the LAP (x-axis) and the PM (y-axis). For the values along the line that bisects the x- and y-axis (magenta line) exactly the same values are measured with the PM and the LAP. The linear fit through the scatterplot (dotted cyan line) is identical to the line that bisects the x- and y-axis within the margin of error. The determined fiber directions are consistent between both systems as the Pearson coefficient is 0.9885. The retardation values show a deviation of < 6.72° from the linear fit within the first confidence interval of the standard deviation (68%). These small deviations are also visible in the differential map (cf. Figure 3A), particularly in brain regions of homogeneous fiber courses, such as the corpus callosum. Larger deviations occurred only in brain regions that contained heterogeneous fiber courses, such as the thalamus and Globus pallidus.

The comparison of the retardation values derived from measurements and evaluated according to Equation (13) with γ = 0.25π, shows significant differences between PM and LAP (cf. Figure 4). Most retardation values derived from the LAP measurement are lower than the ones based on PM measurements, indicated by the fact that most dots are above the line that bisects the x- and y-axis. With increasing retardation values, the absolute difference between the values measured with the LAP and the PM increases as indicated by the higher slope of the fit function in Figure 4B (cyan line). Still, the data is highly correlated which is indicated by a Pearson coefficient of 0.9867. By employing the new analysis method [cf. Equation (13) with γ = 0.259π] to interpret the LAP measurements, higher retardation values are derived. As a result, the fit function through the data points (cyan line) coincides with the line that bisects the x- and y-axis.

Within the first confidence interval of the standard deviation the retardation value based on LAP measurements differs from the downsampled value of the PM by 0.050. Differences in the retardation values are observed particularly in the thalamus, internal capsule and superior corona radiata (Figure 4A).

**Figure 5:**
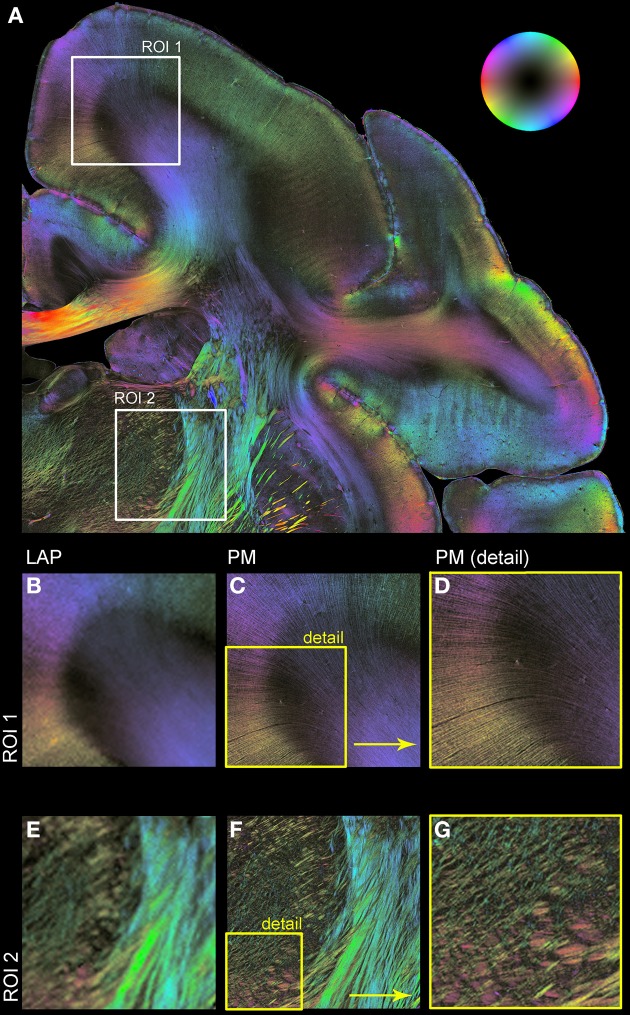
**Comparison of LAP and PM based on FOMs**. Orientation of nerve fibers is color coded according to the color scheme indicated by the bubble in **(A)**. **(A)** Displays a region of interest of a vervet monkey section measured with the PM. For further illustration of the differences and similarities of the LAP **(B,E)** and the PM **(C,D,F,G)** two regions of interest (ROI 1 and ROI 2) are enlarged. **(D,G)** represent an enlargement of **(C,F)** to highlight the high-resolution capabilities of the PM.

Figure 5 shows resulting fiber orientation maps opposing PM and corrected LAP data sets visualized in a HSV color coding scheme (i.e., colors are assigned to distinct fiber orientations). This example demonstrates the general similarity of both measurements (Figures 5B/C,E/F). Large fiber structures, such as fiber bundles in the internal capsule (Figures 5E,F), are contrasted with PM and LAP equally. The PM detects even single fibers in the cerebral cortex and within nuclei (Figures 5C,E), while the LAP FOMs show the orientation in the corresponding brain regions.

### 3.2. Influence of the lateral partial volume effect

**Figure 6:**
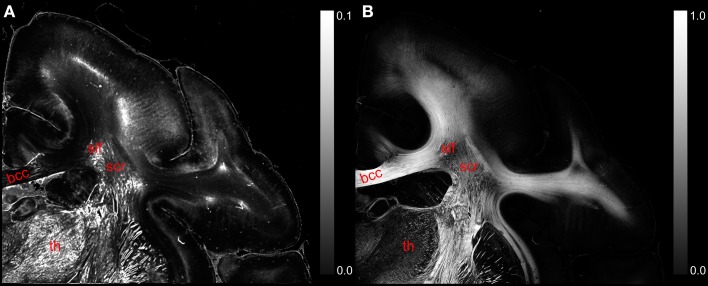
**(A)** Displays a differential map of the downsampled averaged retardation values based on PM measurements and the retardation map determined with the LAP with γ = 0.259π. **(B)** Shows in comparison the retardation map obtained with the PM. (bcc, body of corpus callosum; scr, superior corona radiata; slf, superior longitudinal fasciculus; th, thalamus.)

The comparison of the retardation values measured with the LAP (*r*′_LAP_) and the mean retardation value of the corresponding 48 × 48 PM pixels (${\hat{r}}_{PM}$) revealed that in most cases the averaged PM-value is higher than the LAP-value (cf. Figure 6A). For brain regions with predominantly parallel fibers, the downsampled and the averaged retardation values are similar (e.g., corpus callosum). In contrast, in heterogeneous regions (where fiber populations with different orientations are present), the downsampled retardation values are much lower than average values. Such regions (e.g., thalamus and the superior part of the Corona radiata) appear highlighted in the differential map. In these regions the mixing of different fiber bundles is indicated by a decrease in the signal strength in the retardation map (cf. Figure 6B).

## 4. Discussion

The main purpose of this study was to develop a strategy to combine polarized light imaging (3D-PLI) measurements which were captured at different optical resolutions obtained with different setups. This is an indispensable prerequisite to realize a multiscale approach over two orders of magnitude (in-plane). We accomplished this aim for the in-house developed large-area polarimeter and the polarizing microscope provided by Taorad GmbH, Germany (Axer et al., 2011a). The extracted direction and retardation maps turned out to be well suited to identify sources of differences in the PM and LAP measurements.

This study corroborated that the discrepancy of the peak wavelength of the illumination source and the design wavelength of the used quarter-waveplate has significant impact on the determined retardation signal and consequently on the estimated fiber orientation. Such a discrepancy exists for the LAP, but not for the PM. However, by generalizing the mathematical description of the retardance γ induced by the waveplate and using γ = 0.259π for the LAP setup (instead of γ = 0.25π) we achieved similar retardation values for the LAP and PM measurements by the Fourier-based downsampling. The PM measurements were chosen here as a reference, since (i) previous studies confirmed the optimal matching of illumination wavelength and design wavelength for this setup (Reckfort et al., 2013) and (ii) the optical resolution of the PM is in the same order of magnitude as the targeted axonal diameters (cf. Aboitiz et al., 1992). This approach is superior to the methodology described by Reckfort et al. (2013), where an empirical correction factor was proposed to compensate for the differences between PM and LAP measurements, without considering the extensive effects on fiber orientation estimations (especially in brain regions of complex fiber architecture).

A second key element toward a reliable comparison of PM and LAP data is the downsampling methodology which models the lateral partial volume effects that are very specific to 3D-PLI measurements. Since the measurements provide sinusoidal intensity curves per pixel, combining a neighborhood of pixels into an averaged signal requires the superposition of the individual sinusoidal curves rather than averaging scalar values (i.e., retardation values and direction angles). Consequently, the downsampling procedure was applied to the individual Fourier coefficients to preserve the sinusoidal characteristic of the measurements. Theoretically it is possible to interpolate the LAP images and compare them with the original PM images, however in order to significantly contribute to the neuroanatomical interpretation a sophisticated interpolation such as used in super resolution imaging is necessary. Such a comparison might be interesting and could be considered for a future study.

In addition, it was demonstrated that a simple averaging of the retardation values does not correctly describe the lateral partial volume effects observed in 3D-PLI. Averaging PM retardation values in regions of 48 × 48 pixels led to higher values than measured with the LAP in the corresponding pixel. This is attributed to the fact that the superposition of sinusoidal signals cannot be described adequately in the scalar retardation/direction space (cf. Section 2.6). Nonetheless, the comparison of downsampled PM and measured LAP retardation maps indicated that a differential map of the averaged retardation and the retardation of the averaged sinusoidal curves is a good measure for the magnitude of the lateral partial volume effect expected in LAP measurements. By this means, brain regions of heterogeneous and homogeneous fiber distributions can be classified, which adds valuable information to the estimated fiber orientations. Recent 3D-PLI simulations demonstrated that the extracted fiber orientations of heterogeneous areas are significantly influenced by the relative mixture of fibers Dohmen et al. (2015). The study showed that this is relevant particularly for non-orthogonally crossing fibers where not the predominant fiber direction is estimated, but rather the intermediate fiber direction. Thus, the experimental identification of reliable estimates of fiber orientations will significantly improve the data interpretation. Fiber tractography algorithms running on LAP data sets, for example, will benefit from this information, as they can be guided differently and more reliably through brain regions of known different fiber constellations.

To conclude, the employed polarimetric setups were optimized for investigating nerve fibers at different scales and independently from the optical system properties. We could demonstrate both the mutual and complementary contributions of the two polarimeters. Thus, it is now possible to retrieve comparable results with the large-area polarimeter and the polarization microscope, enabling the investigation of short-range axonal projections and long-distance fiber connections at the sub-millimeter scale. The 3D-PLI data serves as a multiscale description of the human fiber architecture. This consolidates 3D-PLI as a key technology to understand the organization of the human fiber architecture.

## Author contributions

JR substantially contributed to the conception and design of the work as well as to the acquisition, analysis, and interpretation of data for the work. HW substantially contributed to the theoretical derivations of the new approach in this study and the analysis of the measured data. JR and HW drafted co-jointly the manuscript, revised it and both approved the final version to be published. UP substantially contributed to the development of methods, the interpretation of results and revising the manuscript. KZ substantially contributed to the method of tissue preparation and processing, and to the anatomical content of this paper. He further contributed to revision of the manuscript. KA substantially contributed to the anatomical content of this paper, the interpretation of the results and revised the manuscript. MA substantially contributed to the design of the study, the development of methods, the interpretation of results and revising the manuscript. All authors gave their final approval to the submitted version and agreed to be accountable for all aspects of the work.

## Funding

This study was partially supported by the National Institutes of Health under grant agreement no. *R01MH 092311*, by the Helmholtz Association through the Helmholtz Portfolio Theme “Supercomputing and Modeling for the Human Brain,” and by the European Union Seventh Framework Programme (FP7/2007-2013) under grant agreement no. *604102* (Human Brain Project).

### Conflict of interest statement

The authors declare that the research was conducted in the absence of any commercial or financial relationships that could be construed as a potential conflict of interest.

## Footnotes

^1^A more detailed derivation is given in the Supplementary Material.
