# Supplementary material for: A multiscale approach for the reconstruction of the fiber architecture of the human brain based on 3D-PLI
Source: Front Neuroanat. 2015 Sep 3;9:118. doi: 10.3389/fnana.2015.00118 (PMC4558534; doi:10.3389/fnana.2015.00118)
Supplement: Supplementary file 1 [file DataSheet1.PDF]

Supplementary Material: A multiscale approach for the reconstruction of the fiber architecture of the human brain based on 3D-PLI

Julia Reckfort [1,*], Hendrik Wiese [1,*], Uwe Pietrzyk [2,3], Karl Zilles [1,4], Katrin Amunts [1,5] and Markus Axer [1]

[1] *Institute of Neuroscience and Medicine (INM-1), Research Centre Jülich, Germany*
[2] *Institute of Neuroscience and Medicine (INM-4), Research Centre Jülich, Germany*
[3] *Department of Mathematics and Natural Sciences, University of Wuppertal, Germany*
[4] *Department of Psychiatry, Psychotherapy and Psychosomatics, University Hospital, RWTH Aachen University and JARA Translational Brain Medicine, Germany*
[5] *C. and O. Vogt Institute for Brain Research, Heinrich-Heine University Düsseldorf, Germany*

Correspondence*:
Julia Reckfort and Hendrik Wiese
Institute of Neuroscience and Medicine (INM-1), Fiber Architecture, Research Centre Jülich, Leo-Brandt-Strasse, 52428 Jülich, Germany,
j.reckfort@fz-juelich.de, h.wiese@fz-juelich.de

1 SUPPLEMENTARY DATA

As described in Section 2.1 the standard setup consists of a pair of crossed linear polarizers (P_x and P_y) and a waveplate ($\mathrm{M}_{\text{ret}}(\gamma)$). The fast axis of the waveplate is rotated by $\psi = -45\,^\circ$ with respect to the axis of the first linear polarizer). The influence of the optical components regarding the incident light can be described by Jones matrices. The linear polarizers are expressed by the Jones matrices $P_x = \begin{pmatrix} 1 & 0 \\ 0 & 0 \end{pmatrix}$ and $P_y = \begin{pmatrix} 0 & 0 \\ 0 & 1 \end{pmatrix}$ and the waveplate by

$$M_{\text{ret}}(\gamma) = \begin{pmatrix} \cos(\gamma/2) & -\mathrm{i}\sin(\gamma/2) \\ -\mathrm{i}\sin(\gamma/2) & \cos(\gamma/2) \end{pmatrix}, \tag{1}$$

where γ is the induced light retardance of the extraordinary ray (**Jones** (1941)). The influence of the brain tissue can be described by the matrix of an arbitrary waveplate (**Collett** (2009)):

$$M'_{\text{fiber}} = \begin{pmatrix} \cos(\delta/2) + \mathrm{i}\sin(\delta/2)\cos(2\beta) & \mathrm{i}\sin(\delta/2)\sin(2\beta) \\ \mathrm{i}\sin(\delta/2)\sin(2\beta) & \cos(\delta/2) - \mathrm{i}\sin(\delta/2)\cos(2\beta) \end{pmatrix}. \tag{2}$$

During the 3D-PLI measurements the filters are rotated. However, instead of applying the rotation matrices to all three polarization filters, the equivalent case in which the specimen stage is rotated by an angle β

is considered. $\beta = \rho - \varphi$ is the difference between the rotation angle ρ and the in-plane direction of the nerve fibers φ. The phase retardance induced by the brain tissue is described by δ. The light emitted by the employed light source is unpolarized. After the transmission through the first linear polarizer the light is linearly polarized (P_x, cp. **Figure 1**). As it is not possible to describe unpolarized light with the Jones matrix calculus, the Jones vector E_x will be used to describe the linearly polarized light after passing the first linear polarizer. Hence, the influence of the polarimetric setup and the brain tissue is described by:

$$\begin{aligned}\vec{E_T}'(\delta,\beta,\gamma) =& P_y \cdot M'_{\text{fiber}}(\delta,\beta) \cdot M_{\text{ret}}(\gamma) \cdot \vec{E_x} \\ =& \left[\mathrm{i}(\sin(2\beta)\sin(\delta/2)\cos(\gamma/2) - \cos(\delta/2)\sin(\gamma/2)) - \cos(2\beta)\sin(\delta/2)\sin(\gamma/2)\right] E'_x \vec{e}_y.\end{aligned}$$

The measured light intensity corresponds to the absolute square of the electric field vector ($I'_T \propto |\vec{E}'_T|^2$):

$$\begin{aligned}I'_T(\delta,\beta,\gamma) =& \Big(\sin^2(\gamma/2)\cos^2(\delta/2) - \frac{1}{2}\sin(2\beta)\sin(\delta)\sin(\gamma) + \sin^2(2\beta)\sin^2(\delta/2)\cos^2(\gamma/2) \\ &+ \cos^2(2\beta)\sin^2(\delta/2)\sin^2(\gamma/2)\Big) I'_{T0} \\ \overset{(\beta=\varphi-\rho)}{=}& \Big(\sin^2(\gamma/2)\cos^2(\delta/2) + \frac{1}{2}\sin^2(\delta/2) + \frac{1}{2}\sin(\gamma)\sin(\delta)\sin(2\rho)\cos(2\varphi) \\ &- \frac{1}{2}\sin(\gamma)\sin(\delta)\sin(2\varphi)\cos(2\rho) - \frac{1}{2}\sin^2(\delta/2)\cos(4\varphi)\cos(4\rho) \\ &\cdot (1 - 2\sin^2(\gamma/2)) - \frac{1}{2}\sin^2(\delta/2)\sin(4\varphi)(1-\sin^2(\gamma/2))\Big) I'_{T0}. \qquad (3)\end{aligned}$$

Analyzing this intensity profile provides us with an adjusted directive to calculate the fiber orientation accounting for a deviation in the retardance of the waveplate.

REFERENCES

Collett, E. (2009), Field guide to Polarization, volume FG05 (SPIE Field Guides)

Jones, R. (1941), A new calculus for the treatment of optical systems I. Description and discussion of the calculus, *Journal of the Optical Society of America*, 31, 7, 488–493, doi:{10.1364/JOSA.31.000488}
